# Echocardiographic predictors of right ventricular failure following left ventricular assist device implantation: Systematic review and meta-analysis

**DOI:** 10.21542/gcsp.2025.35

**Published:** 2025-08-30

**Authors:** Shruti Rajendra, Yousuf Salmasi, Espeed Khoshbin

**Affiliations:** 1 National Heart & Lung Institute, Imperial College, London, UK; 2 Harefield Hospital, Royal Brompton, and Harefield as part of Guys and St Thomas NHS Trust, London, UK

## Abstract

Introduction:  Right ventricular failure (RVF) is a significant complication following left ventricular assist device (LVAD) implantation, with no universally accepted predictors. This meta-analysis identifies the most reliable echocardiographic predictors.

Methods: OVID Medline was systematically searched for observational studies reporting ten preoperative echocardiographic parameters in patients who did and did not develop RVF post-LVAD. Random-effects meta-analyses were performed. Subgroup analyses and meta-regression assessed the influence of RVF definitions and clinical characteristics on predictive capacity. Logistic regression modeling identified key cutoffs.

Results: Thirty-nine studies involving 2,975 patients were included, with pooled RVF prevalence of 0.30 (0.26–0.34). Higher right ventricular end-diastolic diameter (RVEDD) (SMD: 0.368, *p* < 0.0001, I^2^: 1.73%) and less negative right ventricular free wall strain (RVFWS) (SMD: 0.931, *p* < 0.0001, I^2^: 82.9%) were significant predictors. Higher right ventricular end-diastolic area (RVEDA) was also reliable but weaker (SMD: 0.224, *p* = 0.0282, I^2^: 0.00%). Lower tricuspid annular plane systolic excursion (TAPSE) was a strong predictor (SMD: −0.512, *p* < 0.0001,) but less reliable due to high heterogeneity (I^2^: 86.1%). Subgroup analyses of TAPSE by RVF definition showed modestly reduced heterogeneity (43.7%). Predictive capacity was significantly better in more stable patients (i.e., no inotropes/IABP, higher INTERMACS status). Logistic regression identified increased RVF risk at RVEDD ≥41.5 mm (sensitivity: 50.0%, specificity: 83.3%) and RVFWS ≥ −11.3% (sensitivity: 88.9%, specificity: 88.9%).

Conclusion: RVEDD ≥ 41.5 mm and RVFWS ≥ −11.3% are the strongest, most reliable predictors of RVF post-LVAD. Variations in RVF endpoint definitions only partially explain the observed heterogeneity, while patient characteristics significantly influence predictive accuracy. Future studies should explore subgroup-specific cutoffs.

## Introduction

Left ventricular assist devices (LVADs) have revolutionized the management of advanced heart failure, serving as a critical bridge to heart transplantation, or as destination therapy^1–3^. However, right ventricular failure (RVF)—a severe complication affecting 10–40% of LVAD recipients—remains a significant barrier to improved outcomes^1–3^. The exact pathophysiology of RVF is not fully understood but is thought to result from the sudden restoration of left ventricular function, which increases venous return to the right ventricle (RV), causing it to strain under the sudden increased load, leading to its dysfunction and eventual failure^1–3^. RVF is well recognized as a major cause of poorer outcomes post-LVAD, often necessitating additional interventions such as temporary right ventricular assist device (RVAD) insertion or prolonged inotropic support in intensive care settings^4,5^. Preoperative identification of high-risk patients is important, as it facilitates better patient selection for planned biventricular assist device (BiVAD) implantation, which has demonstrated superior outcomes compared to staged LVAD implantation followed by RVAD insertion^6^. Preoperative identification of high-risk patients enables tailored management. Decision-making with respect to choice of transplantation versus mechanical circulatory support, preoperative optimization (prehabilitation), and postoperative critical care support, potentially improve outcomes^2^.

Echocardiography, a widely available and noninvasive tool, plays a pivotal role in preoperative cardiac assessment. Several single-center observational studies have proposed echocardiographic markers of RV function as potential predictors of RVF post-LVAD implantation^1–3^. Bellavia et al., who conducted one of the earliest comprehensive meta-analyses in this field, identified several significant predictors, yet the clinical applicability was limited by the high heterogeneity observed^7^. Subsequent analyses, such as those by Benedetto et al., which focused on continuous-flow LVADs, and those by Chriqui et al. and Frye et al., which explored novel strain-based echocardiographic markers like right ventricular global longitudinal strain (RVGLS), still faced challenges with heterogeneity, thus reducing the generalizability of the identified predictors^8–10^. Potential sources of the observed heterogeneity include variations in RVF definitions, variations in LVAD technology and surgical practice, and variations in baseline patient characteristics across centers, although the exact impact of these factors remains unclear.

This meta-analysis incorporates a larger pool of studies and hence aims to provide an updated assessment of preoperative right-sided echocardiographic predictors of RVF following LVAD implantation. It addresses the longstanding issue of heterogeneity by using subgroup and meta-regression analyses to evaluate the impact of RVF endpoint definitions and LVAD technology, and explore the predictive value of the identified echocardiographic parameters across specific patient subgroups. Finally, considering the inconsistent findings across studies, this meta-analysis aims to utilize all the available literature to propose cutoffs for the most significant and reliable predictors. Resolving these challenges is essential for enhancing risk stratification and improving outcomes for LVAD recipients.

## Material and Methods

### Search strategy

A systematic literature search was conducted in February 2024 on the OVID Medline database. Records dating from inception through February 13, 2024 were searched using keywords associated with RVF and LVAD, as detailed in Table S1. After excluding duplicate records, titles and abstracts were screened. Articles were included if they were original primary research assessing predictors of RVF post-LVAD. The following were the exclusion criteria: Wrong study type (i.e., case reports, reviews), studies including preplanned BiVAD patients, studies with no full-text availability, pediatric studies (patients aged <18 years), and non-English language publications.

Full texts of the remaining articles were further screened. Articles were included for final analysis if they reported preoperative echocardiographic data for two patient groups: (1) those who developed RVF following LVAD implantation, and (2) those who did not. Articles were also excluded at this stage if they only explored non-echocardiographic predictors or focused solely on patient subgroups with specific comorbidities.

Study quality was assessed using the Newcastle-Ottawa Scale to check for risk of bias. Studies with ≥7/9 responses were considered of sufficient quality to be included in final analysis^11^.

### Study endpoints and data collection

The primary endpoint was RVF following LVAD implantation, as defined by each study. Definitions included:

1. Clinical signs of RV dysfunction;
2. Need for interventions (namely RVAD implantation);
3. Prolonged inotropic support.

Subgroup analyses focused on studies using the most common RVF definition: need for RVAD implantation or inotropes for > 14 days (± prolonged vasodilator or inhaled nitric oxide (iNO) therapy)^12,13^. This definition was selected for its reflection of clinically severe RVF and widespread use in contemporary studies^12,13^. Subgroup analyses of studies using alternative definitions were not performed due to insufficient data. Separate subgroup analyses focused on studies using continuous-flow LVADs only were also performed. Ten preoperative echocardiographic parameters were evaluated, including three functional markers (tricuspid annular plane systolic excursion (TAPSE), right ventricular fractional area change (RVFAC), and right ventricular ejection fraction (RVEF)), four structural markers (right ventricular end-diastolic diameter (RVEDD), right ventricular to left ventricular diameter ratio (RV/LV diameter ratio), right ventricular end-diastolic area (RVEDA), and right ventricular end-systolic area (RVESA)), and three strain markers (right ventricular global longitudinal strain (RVGLS), right ventricular free wall strain (RVFWS), and right ventricular septal longitudinal strain (RVSLS)). These were selected for their frequent reporting and clinical relevance. When multiple values for a parameter were available, basal RVEDD, apical RV/LV ratios, and apical four-chamber views for RVFAC, RVGLS, RVFWS, and RVSLS were used to minimize variation, since these measurements are the most reported.

For each parameter, means, standard deviations, and sample sizes for the RVF and non-RVF groups were extracted. When studies reported medians and interquartile ranges, or medians and ranges, the means and standard deviations were obtained using the following estimations, as proposed in the Cochrane Handbook for Systematic Reviews: (1) Mean = median; (2) Standard deviation = interquartile range/1.35; (3) Standard deviation = range/4^14^.

The following additional data were extracted from each study: gender distribution, mean age, mean BMI, mean preoperative INTERMACS score, the proportions of patients with ischemic cardiomyopathy, receiving continuous-flow LVADs, on preoperative inotropic support, population of patients supported using intra-aortic balloon pump (IABP) therapy, and the proportions of patients with different indications for LVAD therapy such as bridge-to-transplantation, bridge-to-decision, bridge-to-recovery, bridge-to-candidacy, or destination therapy.

### Statistical analyses

All statistical analyses were performed using STATA version BE/18.0. Missing data were managed appropriately by statistical software using listwise deletion, meaning that only studies reporting the relevant parameter were included in each respective analysis. No imputation was performed since this was a meta-analysis of published aggregate level data, where imputing values requires significant assumptions that could introduce bias.

The pooled prevalence of RVF following LVAD implantation across all studies was calculated, followed by subgroup analysis restricted to studies using the most common RVF definition.

Random-effects meta-analyses using the DerSimonian-Laird method estimated overall effect sizes for each echocardiographic parameter. Since all parameters were continuous variables, standardized mean difference (SMD) using Hedge’s g was used. Given the expected heterogeneity among retrospective observational studies, random-effects models were applied. Adjustments for multiple comparisons across the parameters were not performed since the analyses were exploratory in nature. This approach avoids overcorrection and the potential loss of meaningful associations. Subgroup meta-analyses including only studies using the most common RVF definition were performed for parameters with sufficient data (≥15 studies), in order to assess whether endpoint definitions influenced heterogeneity and predictive value.

Heterogeneity was assessed using the I^2^ statistic and Cochrane’s Q test, and categorized by I^2^ values as low (< 25%), moderate (25–50%), high (50–75%), or very high (> 75%), as suggested by Higgins et al. ^15^. Leave-one-out sensitivity analyses were conducted to identify potential outliers.

Meta-regression analyses were performed for parameters with data from ≥15 studies to explore the influence of patient demographics, clinical characteristics, and the year of publication on predictive capacity.

Finally, to estimate optimal cutoff values for each significant predictor, weighted logistic regression modeling was performed using pooled summary-level data from the studies included in this meta-analysis. For each predictor, the RVF and non-RVF groups from each study were treated as individual observations. The group mean was used as the predictor value, RVF status (1 = RVF, 0 = no RVF) was set as the binary outcome, and group sample size was applied as a frequency weight. Logistic regression was used to model the relationship between predictor values and the probability of RVF. Predicted probabilities of RVF were then calculated across a continuous range of predictor values. The optimal cutoff for each parameter was defined as the value corresponding to the maximum Youden’s index (sensitivity + specificity – 1), which balances sensitivity and specificity. Receiver operating characteristic (ROC) analyses were also conducted to assess model discrimination, and the sensitivity and specificity at each optimal cutoff were reported.

**Figure 1.**
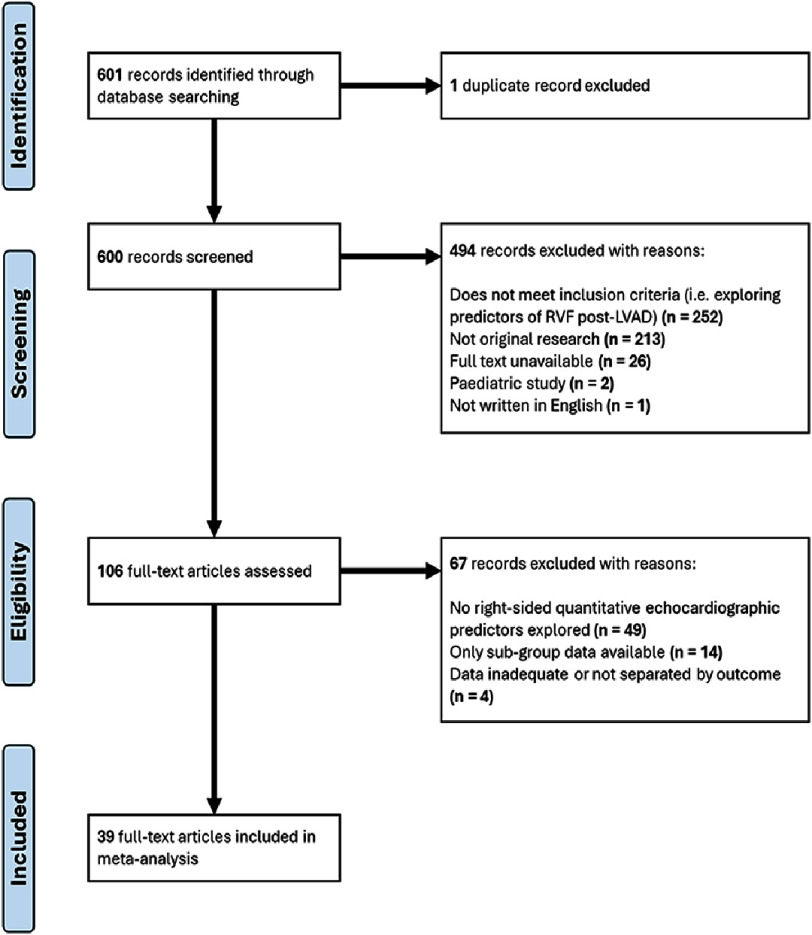
PRISMA flowchart of study selection for meta-analysis.

## Results

### Study selection and characteristics

The initial database search identified 601 records, from which 1 duplicate record was excluded. After titles and abstract screening, 494 were excluded. Full-text screening of the remaining 106 records led to a further 67 records excluded (Figure 1).

A total of 39 articles published between 1999 and 2022 were included in the final meta-analyses. All studies were observational, with 8 using prospectively collected data and the remaining 31 using retrospectively collected data. All but one study were single-center investigations; the exception was a multicenter study. The pooled population consisted of 2,923 patients, of whom 883 (30.2%) developed RVF post-LVAD. The characteristics of the individual studies are summarized in Table 1.

**Table 1:** Summary of study characteristics. Studies that were included in the subgroup analysis as they used the commonest RVF definition (detailed under ‘Methodology’).

**Study, Year of Publication**	**Study design**	**Country**	**Study period**	**Definition of endpoint**	**Sample size (n)**	**RVF post-LVAD (n, %)**
Aissaoui et al., 2015*^13^	Prospective observational single-center	Germany	Nov 2010 to Aug 2011	Need for RVAD OR inotropes for > 14 days	42	24 (57.1)
Alfirevic et al., 2020*^16^	Retrospective observational single-center	USA	Jan 2010 to Dec 2017	Need for RVAD OR inotropes or inhaled vasodilators for > 14 days	86	15 (17.4)
Boegershausen et al., 2017^17^	Retrospective observational single-center	Germany	Jan 2014 to Jan 2016	Need for RVAD OR inotropes or iNO for > 7 days in the presence of CVP > 18 mmHg with CI<2.3 L/min/m^2^ in the absence of LA or PCWP > 18 mmHg, cardiac tamponade, ventricular arrhythmias or pneumothorax	44	12 (28.6)
Bowen et al., 2021^18^	Retrospective observational single-center	Netherlands	2016 to 2019	Need for RVAD OR inotropes > 7 days	25	7 (28.0)
Cacioli et al., 2022*^19^	Retrospective observational single-center	Italy	May 2013 to Aug 2021	Need for RVAD OR inotropes for > 14 days OR iNO for > 48hrs	54	19 (35.2)
Carmona et al., 2020^20^	Retrospective observational single-center	France	Jan 2010 to May 2018	Need for RVAD OR inotropes for > 7 days	68	32 (47.1)
Charisopoulou et al., 2019^21^	Retrospective observational single-center	UK	18 months	Need for RVAD	70	14 (20.0)
Critoph et al., 2015*^22^	Retrospective observational single-center	Australia	Sept 2004 to Jun 2013	Need for RVAD OR inotropes for > 14 days	65	6 (9.23)
Fukamachi et al., 1999^23^	Retrospective observational single-center	USA	Dec 1991 to Dec 1996	Need for RVAD	100	11 (11.0)
Grant et al., 2012*^24^	Retrospective observational single-center	USA	May 2007 to Apr 2011	Need for RVAD OR inotropes for > 14 days	117	47 (40.2)
Gudejko et al., 2019*^25^	Retrospective observational single-center	USA	Mar 2013 to Mar 2016	Need for RVAD OR inotropes or inhaled pulmonary vasodilators for > 14 days	85	28 (32.9)
Gumus et al., 2019^26^	Retrospective observational single-center	Turkey	Jan 2012 to May 2018	Absence of cardiac tamponade within first 48hrs post-surgery AND following: MAP <55mmHg, CVP or RAP > 16mmHg, CI <2L/min/m^2^, requirement of prolonged postimplant inotropes (inotropic score > 20 units), iNO or IV vasodilators > 14 days, need for RVAD or ECMO	57	20 (35.1)
Hennig et al., 2011^27^	Prospective observational single-center	Germany	May 2001 to Dec 2002	2 of following during week 1 post-op: MAP ≤55 mmHg, CVP ≥16 mmHg, mixed venous saturation ≤55%, cardiac index <2 L/min/m^2^, inotropic support score > 20 units or apparent need for mechanical RV support	40	14 (35.0)
Kalogeropoulos et al., 2015*^28^	Retrospective observational single-center	USA	Jan 2008 to Dec 2013	Need for mechanical RV support OR inotropes > 14 days OR pulmonary vasodilators > 48hrs OR multi-organ failure due to RVF	116	37 (31.9)
Kang et al., 2016^29^	Retrospective observational single-center	USA	2010 to 2013	Need for RVAD	83	9 (10.8)
Kapelios et al., 2015^30^	Retrospective observational single-center	Greece	Feb 2006 to Nov 2013	Appearance of persistent signs/symptoms of peripheral vascular congestion (elevated CVP, hepatomegaly, congestion-related pain of RUQ, peripheral edema, ascites, increased BNP) necessitating significant up-titration of diuretics (increase of > 80 mg or 3-fold the initial dose) or positive inotropic agents	20	9 (45.0)
Kato, Chokshi et al., 2012*^31^	Prospective observational single-center	USA	Jan 2002 to May 2010	Need for RVAD OR inotropes for > 14 days or iNO for > 48hrs	61	23 (37.7)
Kato, Maryjane et al., 2012*^32^	Prospective observational single-center	USA	Jan 2007 to Jun 2010	Need for RVAD OR inotropes for > 14 days or iNO for > 48hrs	111	35 (31.5)
Kato et al., 2013*^33^	Prospective observational single-center	USA	Aug 2010 to Feb 2012	Need for RVAD OR inotropic and/or inhaled pulmonary vasodilators (including iNO) for > 14 days	68	24 (35.3)
Kiernan et al., 2015*^34^	Retrospective observational single-center	USA	Jan 2008 to Dec 2011	Need for BiVAD OR inotropes for > 14 days	26	12 (46.2)
Kukucka et al., 2011^35^	Prospective observational single-center	Germany	Jan 2007 to Apr 2009	Need for RVAD OR (absence of cardiac tamponade within first 48hrs post-surgery AND 2 of following: MAP <55mmHg, CVP > 16mmHg, CI <2L/min/m^2^, inotropic score > 20 units)	115	15 (13.0)
Liang et al., 2022*^36^	Retrospective observational single-center	USA	2015 to 2018	Need for RVAD within 30 days OR failure to wean off inotropes, vasopressors or iNO within 14 days	55	28 (50.9)
Lo et al., 2015*^37^	Retrospective observational single-center	Australia	Jun 2003 to Dec 2013	Need for mechanical right heart support OR inotropes for > 14 days or discharged home with it OR iNO for > 48 hrs	101	63 (62.4)
Loforte et al., 2018^38^	Retrospective observational multicenter	Italy	Jan 2006 to Dec 2017	Need for unplanned biVAD	206	71 (34.5)
Magunia et al., 2018*^39^	Retrospective observational single-center	Germany	Oct 2013 to Jul 2017	Need for RVAD OR inotropes for > 14 days	26	5 (19.2)
Montalto et al., 2021^40^	Retrospective observational single-center	Italy	Nov 2015 to Dec 2019	Leftward shift of interventricular septum, MAP <65 mmHg, CVP > 15 mmHg, LVAD flow <3.5 L/m, moderate-severe tricuspid regurgitation, frequent suction events	38	10 (26.3)
Patel et al., 2019*^41^	Retrospective observational single-center	USA	Jan 2008 to Nov 2017	Need for RVAD OR inotropes for > 14 days	46	8 (17.4)
Potapov et al., 2008^42^	Retrospective observational single-center	Germany	Jan 1998 to Apr 2006	2 of following within first 48hrs post-surgery: MAP ≤55mmHg, CVP ≥16mmHg, mixed venous saturation ≤55%, cardiac index <2L/min/m^2^, inotropic support score > 20 units or need for RVAD	54	9 (16.7)
Puwanant et al., 2008*^43^	Retrospective observational single-center	USA	Jan 2004 to Jul 2007	Need for RVAD OR inotropes or pulmonary vasodilators for > 14 days	33	11 (33.3)
Raina et al., 2013*^44^	Retrospective observational single-center	USA	May 2008 to Jun 2011	Need for RVAD OR inotropes for > 14 days	42	16 (38.1)
Raymer et al., 2019*^45^	Retrospective observational single-center	USA	Jun 2008 to Sept 2014	Need for RVAD OR inotropes for > 14 days OR death within 14 days due to RV failure	216	74 (34.3)
Ruiz-Cano et al., 2020^46^	Retrospective observational single-center	Germany	2016 to 2018	CVP > 16mm Hg with CI <2.3 L/min/m^2^ (in absence of elevated PCWP, tamponade, ventricular arrhythmias or pneumothorax) requiring previously unplanned temporary RVAD or iNO or iloprost inhalation and IV inotropes beyond day 14 post-op	80	26 (32.5)
Samura et al., 2019*^47^	Retrospective observational single-center	Japan	Jun 2013 to Oct 2017	Need for RVAD OR inotropes for > 14 days	71	32 (45.1)
Sert et al., 2020*^48^	Retrospective observational single-center	Turkey	Sept 2013 to Sept 2016	Need for RVAD OR inotropes for > 14 days	71	21 (29.6)
Silverton et al., 2018*^49^	Retrospective observational single-center	USA	Apr 2010 to Dec 2016	Need for RVAD OR inotropes for > 14 days	100	19 (19.0)
Stricagnoli et al., 2021^50^	Prospective observational single-center	Italy	Jul 2009 to Feb 2019	Elevated CVP with depressed CI (<2 L/min/m^2^) in the absence of elevated PCWP (<18 mmHg) OR need for RVAD OR prolonged (4 days–1 week) inotropes or iNO	38	8 (21.1)
Terzic et al., 2022^51^	Prospective observational single-center	Serbia	Jun 2013 to Mar 2021	7 days of support and 2 criteria: (1) records of elevated CVP by direct measurement (CVP or RAP > 16 mmHg) or dilated IVC without any inspiratory variation or elevated jugular venous distension; (2) manifestations of elevated CVP characterized by peripheral edema (> 2 either new or unresolved), presence of ascites or palpable hepatomegaly (physical examination or diagnostic imaging), or laboratory evidence of hepatic (total bilirubin > 34 µmol/L) or renal dysfunction (creatinine > 176 µmol/L)	92	20 (21.7)
Valente et al., 2022*^52^	Retrospective observational single-center	Belgium	Feb 2011 to Feb 2020	Need for RVAD OR inotropes for > 14 days OR iNO for > 48hrs	92	24 (26.1)
Vivo et al., 2013*^53^	Retrospective observational single-center	USA	Jan 2004 to Jul 2011	Need for RVAD OR inotropes for > 14 days	109	25 (22.9)

**Notes.**

iNO inhaled nitric oxide CVP central venous pressure CI cardiac index LA left atrium PCWP pulmonary capillary wedge pressure MAP mean arterial pressure RAP right atrial pressure IVC inferior vena cava BNP B-type natriuretic peptide ECMO extracorporeal membrane oxygenation RUQ right upper quadrant

### Meta-analyses

The overall pooled prevalence of RVF post-LVAD was 0.30 (95% CI [0.26–0.34]), with very high heterogeneity (*I*^2^ = 85.4%, *p* < 0.01) (Figure S1A).

Subgroup analysis of studies using the most common RVF definition (detailed under ’Methodology’) yielded a similar pooled prevalence of 0.33 (95% CI [0.28–0.39]), with similarly very high heterogeneity (*I*^2^ = 85.3%, *p* < 0.01) (Figure S1B).

Meta-analyses were conducted for all ten echocardiographic parameters. Effect sizes, *p*-values, and measures of heterogeneity for both the overall and subgroup meta-analyses are summarized in Table 2.

**Table 2:** Summary of results from primary meta-analyses, results after excluding outliers, and subgroup meta-analyses [(1) only including studies using the commonest RVF definition; (2) only including studies using continuous-flow LVADs], exploring associations between preoperative right-sided echocardiographic parameters and the occurrence of RVF post-LVAD.

**Parameter**	**Analysis**	**No. of studies**	**SMD (95% CI)**	**p-value**	**I^2^**	**(Cochrane’s Q) *p*-value**
**2D echocardiography**						
**TAPSE**	All studies	33	−0.512 (−0.754 to −0.269)	<0.0001	86.1	<0.01
	Subgroup (commonest RVF definition studies only)	21	−0.326 (−0.471 to −0.181)	<0.0001	43.7	0.02
	Subgroup (continuous-flow studies only)	29	−0.530 (−0.796 to −0.263)	0.0001	87	<0.01
**RV FAC**	All studies	25	−0.498 (−0.749 to −0.247)	0.0001	80.1	<0.01
	Outlier study excluded	24	−0.342 (−0.488 to −0.196)	<0.0001	41	0.0198
	Subgroup (commonest RVF definition studies only)	16	−0.367 (−0.542 to −0.191)	<0.0001	41.9	0.04
	Subgroup (continuous-flow studies only)	20	−0.516 (−0.832 to −0.200)	0.0014	84	<0.01
**RV/LV ratio**	All studies	15	0.566 (0.194 to 0.938)	0.0029	84.7	<0.01
	Subgroup (commonest RVF definition studies only)	10	0.414 (0.148 to 0.679)	0.0023	56.5	0.014
	Subgroup (continuous-flow studies only)	14	0.594 (0.197 to 0.990)	0.0034	85.7	<0.01
**RVEF**	All studies	8	−0.803 (−1.36 to −0.245)	0.0048	80.1	<0.01
**RVEDD**	All studies	12	0.368 (0.203 to 0.533)	<0.0001	1.73	0.43
**RVEDA**	All studies	6	0.224 (0.024 to 0.424)	0.0282	0	0.912
**RVESA**	All studies	4	0.184 (−0.040 to 0.407)	0.107	0	0.738
**Strain echocardiography**						
**RVGLS**	All studies	8	0.847 (0.231 to 1.46)	0.0071	89.4	<0.01
	Outlier study excluded	7	0.461 (0.197 to 0.724)	0.0006	40	0.125
**RVFWS**	All studies	9	0.931 (0.432 to 1.43)	0.0003	82.9	<0.01
**RVSLS**	All studies	4	1.38 (−0.081 to 2.84)	0.0641	94.1	<0.01
	Outlier study excluded	3	0.541 (0.031 to 1.05)	0.0378	43.5	0.17

**Notes.**

SMD standardised mean difference TAPSE Tricuspid Annular Plane Systolic Excursion RVFAC Right Ventricular Fractional Area Change RV/LV ratio Right Ventricular to Left Ventricular Diameter Ratio RVEDD Right Ventricular End-Diastolic Diameter RVEF Right Ventricular Ejection Fraction RVEDA Right Ventricular End-Diastolic Area RVESA Right Ventricular End-Systolic Area RVGLS Right Ventricular Global Longitudinal Strain RVFWS Right Ventricular Free-Wall Strain RVSLS Right Ventricular Septal Longitudinal Strain

### Functional echocardiographic predictors

**Figure 2.**
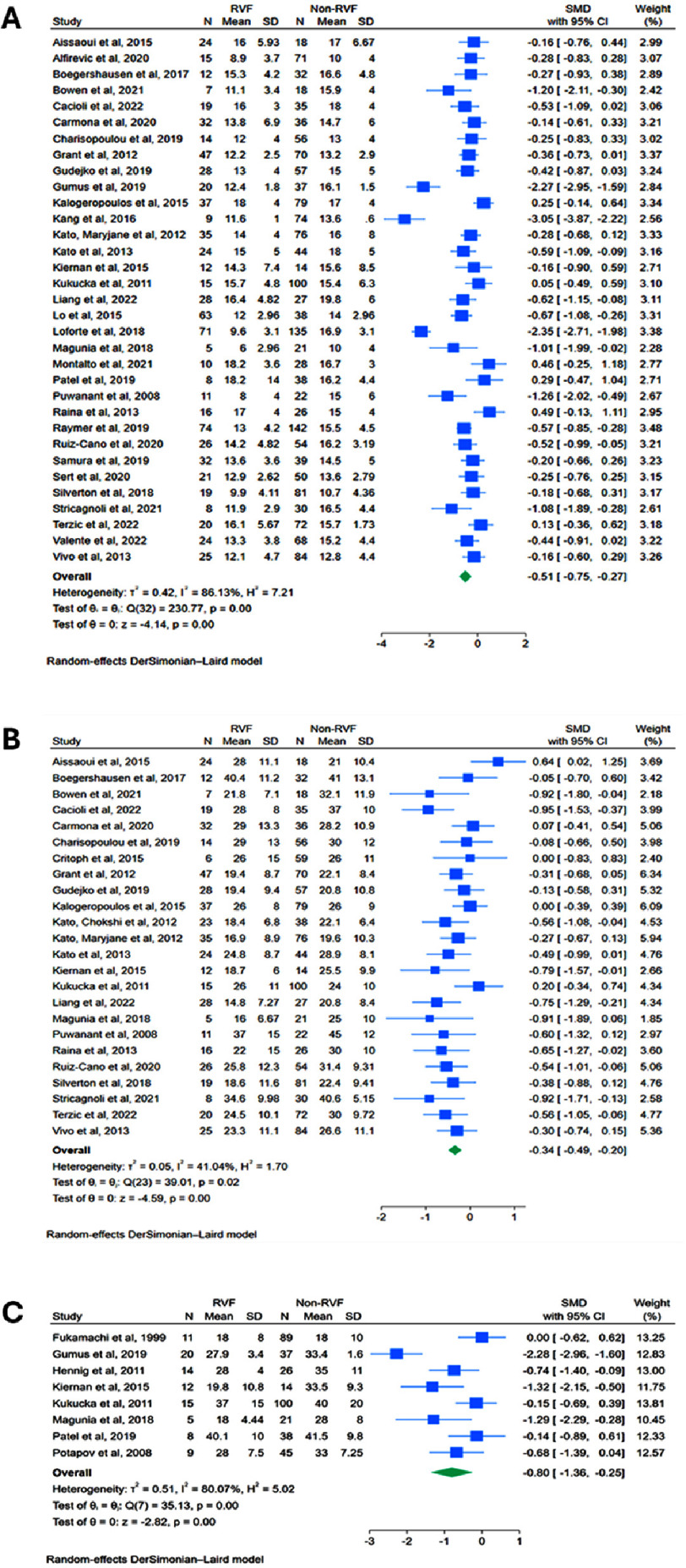
Functional echocardiographic predictors of RVF post-LVAD: Forest plots of meta-analyses comparing preoperative values between RVF versus non-RVF patients: (A) TAPSE; (B) RVFAC*; (C) RVEF. *excludes an outlier study. SMD = standardised mean difference; TAPSE = Tricuspid Annular Plane Systolic Excursion; RVFAC = Right Ventricular Fractional Area Change; RVEF = Right Ventricular Ejection Fraction.

Preoperative TAPSE was reported in 33 studies. Meta-analysis revealed significantly lower TAPSE in patients who developed RVF post-LVAD compared to those who did not (SMD: −0.512, 95% CI: −0.754 to −0.269, *p* < 0.0001) (Figure 2A). Heterogeneity was very high (*I*^2^ = 86.1%, *p* < 0.01), though leave-one-out sensitivity analysis identified no clear outliers (Figure S2A). Subgroup analysis including only the 21 studies using the most common RVF definition yielded a significant but slightly attenuated effect size (SMD: −0.326, 95% CI: −0.471 to −0.181, *p* < 0.0001) (Figure S3A). Heterogeneity reduced substantially to a moderate level (*I*^2^ = 43.7%, *p* = 0.02). A separate subgroup analysis of the 29 studies that involved only continuous-flow LVADs also yielded a significant effect size (SMD: −0.530, 95% CI: −0.796 to −0.263, *p* = 0.0001), and heterogeneity remained very high (*I*^2^ = 87.0%, *p* < 0.01) (Figure S4A).

Preoperative RVFAC, reported in 25 studies, was significantly lower in patients that developed RVF (SMD: −0.498, 95% CI: −0.749 to −0.247, *p* = 0.0001) (Figure S5). Heterogeneity was very high (*I*^2^ = 80.1%, *p* < 0.01). Leave-one-out sensitivity analysis identified Gumus et al. as a potential outlier as it showed an abnormally lower RVFAC in the RVF group compared to other studies, which may be due to the much younger patient cohort (mean age of 39.8 years) (Figure S2B))^26^. Results remained consistent even after excluding this study, with a significant though attenuated effect size (SMD: −0.342, 95% CI: −0.488 to −0.196, *p* < 0.0001), and heterogeneity reduced to a moderate level (*I*^2^ = 41.0%, *p* = 0.0198) (Figure 2B). Subgroup analysis including only the 16 studies using the most common RVF definition showed consistent results (SMD: −0.367, 95% CI: −0.542 to −0.191, *p* < 0.0001), with heterogeneity remaining moderate (*I*^2^ = 41.9%, *p* = 0.04) (Figure S3B). A separate subgroup analysis of the 20 studies that involved only continuous-flow LVADs also yielded a significant effect size (SMD: −0.516, 95% CI: −0.832 to −0.200, *p* = 0.0014), and heterogeneity remained very high (*I*^2^ = 84.0%, *p* < 0.01) (Figure S4B).

Preoperative right ventricular ejection fraction (RVEF), reported in 8 studies, was significantly lower in patients that developed RVF (SMD: −0.803, 95% CI: −1.36 to −0.245, *p* = 0.0048) **(Figure 2C)**. Heterogeneity was very high (*I*^2^ = 80.1%, *p* < 0.01), though leave-one-out sensitivity analysis showed no clear outliers (Figure S2C).

### Structural echocardiographic predictors

**Figure 3.**
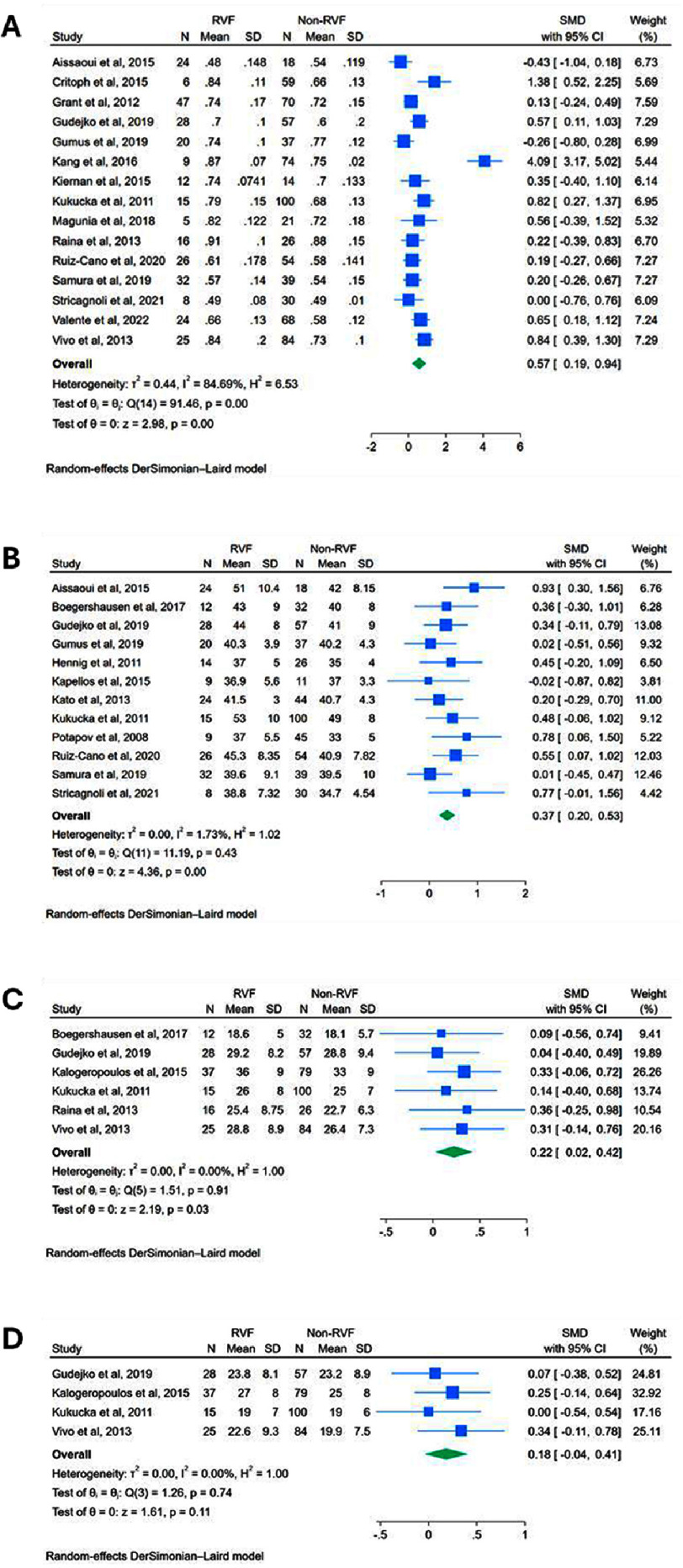
Structural echocardiographic predictors of RVF post-LVAD: Forest plots of meta-analyses comparing preoperative values between RVF versus non-RVF patients: **(A)** RV/LV diameter ratio; **(B)** RVEDD; **(C)** RVEDA; **(D)** RVESA. RV/LV ratio = right ventricular to left ventricular diameter ratio; RVEDD = right ventricular end-diastolic diameter; RVEDA = right ventricular end-diastolic area; RVESA = right ventricular end-systolic area.

Preoperative RV/LV diameter ratio, reported in 15 studies, was significantly higher in patients who developed RVF (SMD: 0.566, 95% CI: 0.194 to 0.938, *p* = 0.0029) (Figure 3A). Heterogeneity was very high (*I*^2^ = 84.7%, *p* < 0.01). Leave-one-out sensitivity analysis identified no clear outliers (Figure S2D). Subgroup analysis of the 10 studies using the most common RVF definition showed consistent results with a slightly stronger effect size (SMD: 0.414, 95% CI: 0.148 to 0.679, *p* = 0.0023), and though heterogeneity decreased, it remained high (*I*^2^ = 56.5%, *p* = 0.014) (Figure S3C). A separate subgroup analysis of the 14 studies that involved only continuous-flow LVADs also yielded a significant effect size (SMD: 0.594, 95% CI: 0.197 to 0.990, *p* = 0.0034), and heterogeneity remained very high (*I*^2^ = 85.7%, *p* < 0.01) (Figure S4C).

**Figure 4.**
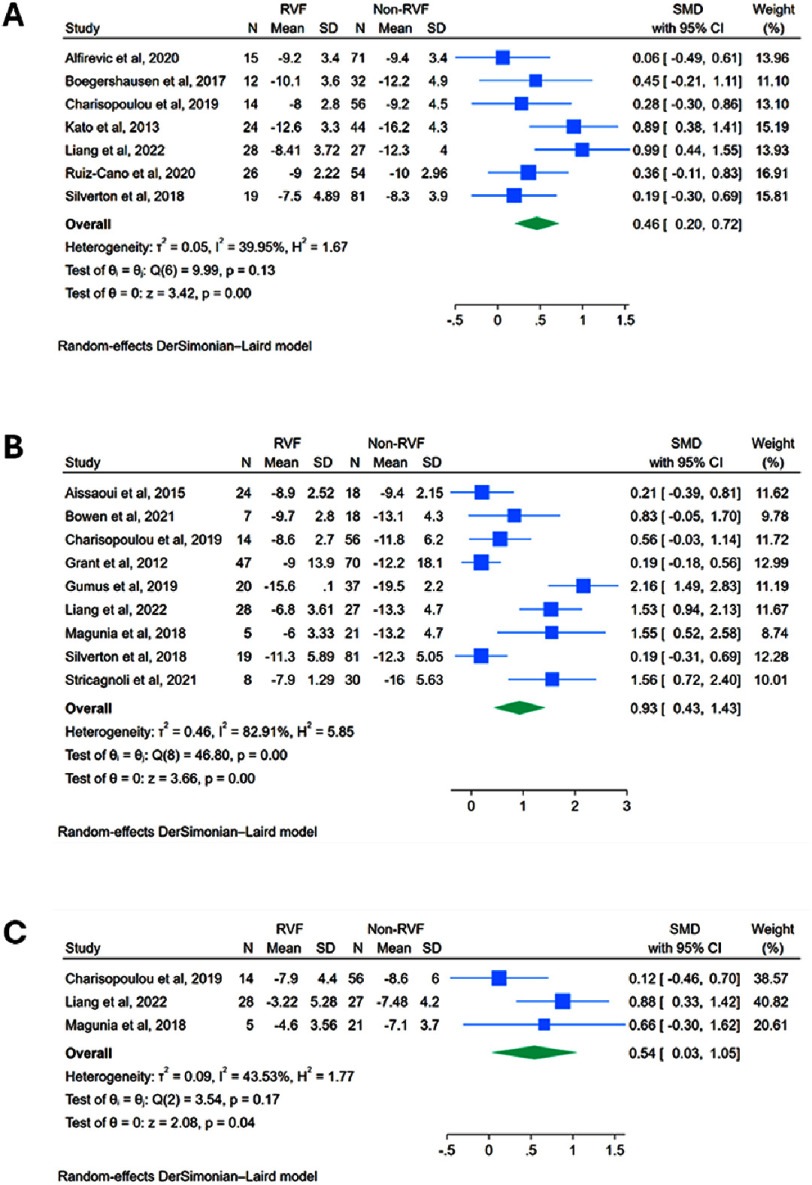
Strain echocardiographic predictors of RVF post-LVAD: Forest plots of meta-analyses comparing preoperative values between RVF versus non-RVF patients: **(A)** RVGLS*; **(B)** RVFWS; **(C)** RVSLS*. *excludes an outlier study. RVGLS = Right Ventricular Global Longitudinal Strain; RVFWS = Right Ventricular Free-Wall Strain; RVSLS = Right Ventricular Septal Longitudinal Strain.

Preoperative RVEDD, reported in 12 studies, was significantly higher in patients who developed RVF (SMD: 0.368, 95% CI: 0.203 to 0.533, *p* < 0.0001) (Figure 3B). There was no significant heterogeneity (*I*^2^ = 1.73%, *p* = 0.43), and leave-one-out sensitivity analysis showed no clear outliers  (Figure S2E).

Preoperative right ventricular end-diastolic area (RVEDA), reported in 6 studies, was significantly higher in patients that developed RVF (SMD: 0.224, 95% CI: 0.024 to 0.424, *p* = 0.0282) with no heterogeneity (*I*^2^ = 0.00%, *p* = 0.912) (Figure 3C). Preoperative right ventricular end-systolic area (RVESA), reported in 4 studies, showed no significant differences between the RVF and non-RVF groups (SMD: 0.184, 95% CI: −0.040 to 0.407, *p* = 0.107) with no significant heterogeneity (*I*^2^ = 0.00%, *p* = 0.738) (Figure 3D). Leave-one-out sensitivity analyses showed no clear outliers for either parameter (Figures S2F–G).

### Strain echocardiographic predictors

Preoperative RV global longitudinal strain (RVGLS), reported in 8 studies, was significantly less negative in patients that developed RVF (SMD: 0.847, 95% CI: 0.231 to 1.46, *p* = 0.0071) (Figure S6A). Heterogeneity was very high (*I*^2^ = 89.4%, *p* < 0.01). Leave-one-out sensitivity analysis identified Gumus et al. as a potential outlier as it showed an abnormally greater difference in RVGLS between the RVF and non-RVF groups compared to other studies (Figure S2H)^26^. Results remained consistent even after excluding this study, with a significant though attenuated effect size (SMD: 0.461, 95% CI: 0.197 to 0.724, *p* = 0.0006), and heterogeneity reduced to a moderate level (*I*^2^ = 40.0%, *p* = 0.125) **(Figure 4A)**.

Preoperative RV free wall longitudinal strain (RVFWS), reported in 9 studies, was significantly less negative in patients that developed RVF (SMD: 0.931, 95% CI: 0.432 to 1.43, *p* = 0.0003) (Figure 4B). Heterogeneity was very high (*I*^2^ = 82.9%, *p* < 0.01), though leave-one-out sensitivity analysis showed no clear outliers (Figure S2I).

Preoperative RV septal longitudinal strain (RVSLS), reported in 4 studies, was significantly less negative in patients that developed RVF (SMD: 1.38, 95% CI: −0.081 to 2.84, *p* = 0.0641) (Figure S6B). Heterogeneity was very high (*I*^2^ = 94.1%, *p* < 0.01). Leave-one-out sensitivity analysis identified Gumus et al. as a potential outlier as it showed an abnormally greater difference in RVSLS between the RVF and non-RVF groups compared to other studies (Figure S2J)^26^. Results remained consistent even after excluding this study, with a significant though attenuated effect size (SMD: 0.541, 95% CI: 0.031 to 1.05, *p* = 0.0378), and heterogeneity reduced to a moderate level (*I*^2^ = 43.5%, *p* = 0.170) (Figure 4C).

### Meta-regression analyses

Given the moderate-to-high heterogeneity observed across many parameters, meta-regression analyses were performed to explore how patient characteristics and clinical factors influenced the predictive ability of three echocardiographic parameters: TAPSE, RVFAC, and RV/LV diameter ratio (Table 3). Lower TAPSE values demonstrated stronger predictive ability for RVF in studies that included fewer patients receiving preoperative IV inotropes (meta-regression coefficient = 0.0163, *p* = 0.044) (Figure S7A) and in studies with a higher proportion of patients whose LVAD indication was bridge-to-candidacy (meta-regression coefficient = −0.0288, *p* = 0.006) (Figure S7B). Lower RVFAC values were more predictive of RVF in studies with a smaller proportion of patients on preoperative intra-aortic balloon pump (IABP) support (meta-regression coefficient = 0.0175, *p* = 0.033) and in patient cohorts characterized by higher (i.e., more stable) preoperative INTERMACS status (meta-regression coefficient = −0.357, *p* = 0.033) (Figure S7C–S7D). Higher RV/LV diameter ratio was more predictive of RVF in studies with a greater proportion of bridge-to-candidacy patients (meta-regression coefficient = 0.217, *p* = 0.001), fewer bridge-to-transplantation patients (meta-regression coefficient = −0.0379, *p* = 0.015), and a smaller proportion of patients receiving preoperative IV inotropes (meta-regression = −0.0432, *p* = 0.001)  (Figure S7E–G). Other covariates examined, including age, gender, BMI, and LVAD type, showed no significant association with the predictive value of TAPSE, RVFAC, or RV/LV diameter ratio. Meta-regression analyses were not conducted for the remaining predictors due to an insufficient number of studies to allow for reliable interpretation.

**Table 3:** Summary of meta-regression results to assess the impact of covariates on the association between key pre-operative echocardiographic parameters and the development of RVF post-LVAD. N/A refers to analyses that were omitted by the statistical software due to collinearity.

**Predictors**	**Covariates**	**Coefficient (95% CI)**	**p-value**
TAPSE	Age	0.00563 (–0.0402 to 0.0514)	0.810
	Female gender	0.0120 (–0.00209 to 0.0260)	0.095
	BMI	–0.101 (–0.305 to 0.104)	0.335
	Ischaemic aetiology	0.00342 (–0.0151 to 0.0219)	0.717
	Continuous flow LVADs	–0.00162 (–0.0206 to 0.0173)	0.867
	Bridge-to-transplantation indication	0.00441 (–0.0122 to 0.0210)	0.602
	Destination therapy indication	–0.00635 (–0.0324 to 0.0197)	0.632
	Bridge-to-decision indication	0.0173 (–0.0322 to 0.0668)	0.494
	Bridge-to-recovery indication	0.00445 (–0.0569 to 0.0658)	0.887
	**Bridge-to-candidacy indication**	**–0.0288 (–0.0494 to –0.00814)**	**0.006**
	Pre-op INTERMACS status	–0.148 (–0.459 to 0.162)	0.349
	**Pre-op IV inotropes**	**0.0163 (0.000405 to 0.0321)**	**0.044**
	Pre-op IABP	–0.00425 (–0.0344 to 0.0259)	0.782
	Year of publication	–0.00628 (–0.0739 to 0.0614)	0.856
RVFAC	Age	−0.0280 (−0.0616 to 0.00559)	0.102
	Female gender	−0.00362 (−0.0112 to 0.00398)	0.350
	BMI	0.248 (−0.167 to 0.662)	0.242
	Ischaemic aetiology	0.00339 (−0.00792 to 0.0147)	0.557
	Continuous flow LVADs	0.00274 (−0.00695 to 0.0124)	0.579
	Bridge-to-transplantation indication	0.00359 (−0.00908 to 0.0163)	0.578
	Destination therapy indication	−0.00374 (−0.0178 to 0.0103)	0.601
	Bridge-to-decision indication	−0.0356 (−0.0809 to 0.00971)	0.124
	Bridge-to-recovery indication	0.00369 (−0.0206 to 0.0280)	0.766
	Bridge-to-candidacy indication	0.0209 (−0.0614 to 0.103)	0.619
	**Pre-op INTERMACS status**	**−0.357 (−0.685 to −0.0287)**	**0.033**
	Pre-op IV inotropes	0.00639 (−0.00259 to 0.0154)	0.163
	**Pre-op IABP**	**0.0175 (0.00139 to 0.0337)**	**0.033**
	Year of publication	−0.0258 (−0.0622 to 0.0106)	0.165
RV/LV diameter ratio	Age	0.0254 (–0.0373 to 0.0882)	0.426
	Female gender	0.000786 (–0.0246 to 0.0262)	0.952
	BMI	0.0986 (–0.0966 to 0.294)	0.322
	Ischaemic aetiology	0.00933 (–0.0204 to 0.0390)	0.538
	Continuous flow LVADs	0.0128 (–0.0398 to 0.0654)	0.633
	**Bridge-to-transplantation indication**	**–0.0379 (–0.0685 to –0.00737)**	**0.015**
	Destination therapy indication	0.0386 (–0.00191 to 0.0792)	0.062
	Bridge-to-decision indication	0.152 (–0.289 to 0.594)	0.499
	Bridge-to-recovery indication	N/A	N/A
	**Bridge-to-candidacy indication**	**0.217 (0.0941 to 0.340)**	**0.001**
	Pre-op INTERMACS status	–0.0525 (–0.690 to 0.584)	0.872
	**Pre-op IV inotropes**	**–0.0432 (–0.0691 to –0.0172)**	**0.001**
	Pre-op IABP	–0.0206 (–0.0763 to 0.0350)	0.468
	Year of publication	–0.0359 (–0.151 to 0.0790)	0.540

**Notes.**

TAPSE Tricuspid Annular Plane Systolic Excursion RVFAC Right Ventricular Fractional Area Change RV/LV ratio Right Ventricular to Left Ventricular Diameter Ratio

Finally, meta-regression analyses using the year of publication as a moderator revealed no significant impact on the predictive value of TAPSE (coefficient = −0.00628, *p* = 0.856), RVFAC (coefficient = −0.0258, *p* = 0.165), or RV/LV diameter ratio (coefficient = −0.0359, *p* = 0.540), indicating stable predictive associations despite evolving LVAD technologies and surgical practices over time  (Figures S8A–C).

### Echocardiographic thresholds for stratifying RVF risk

Threshold analysis identified optimal cutoff values for the echocardiographic parameters that were identified as significant predictors of RVF in this meta-analysis. Optimal thresholds, along with their sensitivity, specificity, and accuracy (% of correctly classified patients), are summarized in Table 4.

**Table 4:** Summary of optimal cutoffs for various echocardiographic predictors of RVF post-LVAD. Cut-offs were estimated using logistic regression modelling based on pooled study-level data. For each parameter, the optimal threshold, and the corresponding sensitivity, specificity and overall percentage of correctly classified patients are reported.

**Parameter**	**Optimal Cutoff**	**Sensitivity**	**Specificity**	**Correctly Classified**
**TAPSE**	<14.3 mm	66.7%	72.7%	69.7%
**RVFAC**	<19.4%	33.3%	100.0%	67.4%
**RV/LV ratio**	≥0.740	60.0%	80.0%	70.0%
**RVEDD**	≥41.5 mm	50.0%	83.3%	66.7%
**RVEF**	<28.0%	75.0%	75.0%	75.0%
**RVEDA**	≥25.4 cm^2^	83.3%	50.0%	66.7%
**RVGLS**	≥–11.0%	87.5%	50.0%	68.8%
**RVFWS**	≥–11.3%	88.9%	88.9%	88.9%
**RVSLS**	≥–4.60%	50.0%	100.0%	75.0%

**Notes.**

TAPSE Tricuspid Annular Plane Systolic Excursion RVFAC Right Ventricular Fractional Area Change RV/LV ratio Right Ventricular to Left Ventricular Diameter Ratio RVEDD Right Ventricular End-Diastolic Diameter RVEF Right Ventricular Ejection Fraction RVEDA Right Ventricular End-Diastolic Area RVESA Right Ventricular End-Systolic Area RVGLS Right Ventricular Global Longitudinal Strain RVFWS Right Ventricular Free-Wall Strain RVSLS Right Ventricular Septal Longitudinal Strain

The parameter with the strongest discriminatory performance was RVFWS, where a cutoff of ≥−11.3% provided the highest sensitivity (88.9%) and specificity (88.9%). Other markers demonstrating robust discriminatory power included RVEF < 28.0% (sensitivity = 75.0%, specificity = 75.0%), TAPSE <14.3 mm (sensitivity = 66.7%, specificity = 72.7%), and RV/LV diameter ratio ≥0.740 (sensitivity = 60.0%, specificity = 80.0%) (Table 4). Discriminatory performance was consistent even when analysis was restricted to studies employing the most common RVF definition.

RVEDD ≥41.5 mm and RVSLS ≥−4.60% demonstrated high specificity of 83.3% and 100% respectively, but moderate sensitivity of 50% in both. In contrast, RVEDA ≥ 25.4 cm^2^ and RVGLS ≥ −11.0% offered high sensitivity of 83.3% and 87.5% respectively, though specificity was moderate (50.0% for both) (Table 4).

### Publication bias and study quality

Funnel plots and Egger’s test were used to assess publication bias for each parameter (Table S2, Figure S9). Most parameters showed no significant evidence of publication bias, except for RVFAC (Egger’s test, *p* < 0.0001), RV/LV diameter ratio (Egger’s test, *p* = 0.0123), and RVGLS (Egger’s test, *p* < 0.0001). However, once the single outlier study Gumus et al. was removed, there was no longer any evidence of publication bias for RVFAC or RVGLS (Egger’s test, *p* = 0.0894 and *p* = 0.9530 respectively)^26^.

Study quality was assessed using the Newcastle-Ottawa Scale (Table S3)^11^. All included studies met the quality threshold of ≥7/9. Commonly noted quality limitations across the studies included the insufficient reporting of missing data.

## Discussion

This meta-analysis provides an updated evaluation of preoperative echocardiographic predictors of RVF following LVAD implantation, a serious complication that significantly impacts patient outcomes. Our analysis identifies the strongest and most reliable echocardiographic predictors, explores sources of heterogeneity seen in previous studies, and proposes optimal echocardiographic thresholds for risk stratification.

### Key echocardiographic predictors

All echocardiographic parameters assessed demonstrated significant associations with RVF post-LVAD, with the sole exception of RVESA, which did not reach significance.

Among conventional 2D echocardiographic parameters, RVEDD emerged as the most reliable predictor of RVF. Higher RVEDD demonstrated a strong association with RVF (SMD: 0.368, *p* < 0.0001) with no significant heterogeneity, consistent with Bellavia et al., who reported a similar effect size (SMD: 0.31, *p* < 0.01)^7^. Despite its predictive strength, RVEDD remains underreported, underscoring the need for broader adoption in research and clinical practice. RVEDA is also a reliable predictor with no significant heterogeneity between studies, though limited by its more modest effect size (SMD = 0.224).

While RVEF had the largest effect size among the 2D echocardiographic measures (SMD: −0.803), substantial heterogeneity (*I*^2^ = 80.1%) that persisted despite sensitivity analysis diminishes its reliability for widespread clinical risk stratification. We also found that TAPSE was a significant predictor, though it also showed substantial heterogeneity. Unlike Bellavia et al., who concluded TAPSE was non-significant, our results align with Chriqui et al. and Benedetto et al., suggesting TAPSE may be a stronger marker than previously thought. However, its heterogeneity reduces its standalone clinical utility^7–9^. Other parameters, including RVFAC and RV/LV diameter ratio, showed significant associations with RVF but were similarly limited by high heterogeneity and comparatively moderate effect sizes, aligning with previous findings by Bellavia et al. and Benedetto et al.^7,8^.

Strain-based parameters emerged as the most powerful echocardiographic predictors of RVF, demonstrating both the largest effect sizes and the strongest discriminative accuracy. Among these, RVFWS showed the largest effect size (SMD = 0.931), followed by RVGLS and RVSLS where values were significantly less negative in patients who developed RVF. Our findings closely align with those reported by Frye et al., whose strain-focused meta-analysis similarly identified all three markers to be significant predictors^10^. However, both our analyses and those by Frye et al. reported very high heterogeneity (I^2^ = 83–94%), which may limit their translation into clinical practice^10^. This persistent heterogeneity is likely due to differences in image acquisition, processing software, and variability in imaging planes (e.g., apical four-chamber versus RV focused views). Due to the limited number of included studies, detailed subgroup or meta-regression analyses to explore the sources of variability could not be reliably conducted for these parameters. As strain imaging becomes more widespread, future meta-analyses incorporating standardized acquisition and processing protocols will be better positioned to clarify whether specific strain subtypes or techniques offer superior predictive utility.

Beyond statistical significance, threshold analysis identified RVFWS and RVEDD as the most clinically actionable parameters. RVFWS ≥ −11.3% demonstrated excellent discriminatory performance, with both sensitivity and specificity at 88.9%, while RVEDD ≥ 41.5 mm had 83.3% specificity and 50.0% sensitivity. However, these thresholds should be interpreted with caution, since they were derived from pooled study-level means without accounting for within-group variability. This limitation is particularly relevant given that parameters with higher SMDs often appear to have better sensitivity and specificity by nature of pooled analysis^54^. In contrast, RVFAC and RVEDA demonstrated more modest effect sizes and their cutoffs demonstrated high specificity but lower sensitivity. These markers may be more useful for ruling out RVF but risk missing a considerable number of true cases. Such trade-offs reflect the broader challenge of preoperative risk stratification: no single parameter offers an ideal balance of sensitivity and specificity across all patient groups. Together, these findings reinforce that the predictive utility of echocardiographic parameters is highly context-dependent. In clinical practice, synthesizing multiple parameters—rather than relying on a single cutoff—is likely to yield more reliable and generalizable assessments of RVF risk.

### Sources of variability and subgroup-specific insights

Another key objective of this study was to assess whether variability in RVF endpoint definitions contributed to the heterogeneity observed in previous studies. However, subgroup analyses restricted to studies using the most common RVF definition revealed no reduction in heterogeneity for pooled RVF prevalence and only minimal-to-modest reductions in effect sizes for the echocardiographic parameters tested. Similarly, threshold analysis showed negligible differences in the optimum cutoffs, when analysis included all studies compared to only studies using the most common RVF definition. This raises two key issues. Firstly, it may suggest that despite attempts to standardize RVF definitions, inconsistencies in their practical application across centers may still contribute to variability in research results. For example, the most common RVF definition that we used for subgroup analysis includes the need for inotropic support for > 14 days post-LVAD implantation^12^. However, centers may differ in: (1) which drugs are classified as inotropes, (2) whether any dosage of inotropes qualifies as inotropic support, and (3) the dosage thresholds required for this classification. Such discrepancies are underreported and likely account for the persistent heterogeneity observed, even among studies supposedly using the same endpoint definition.

Secondly, these findings suggest that variations in RVF definitions explain only part of the observed heterogeneity, and are unlikely to be the sole driver of the heterogeneity. Demographic and clinical differences across study populations likely play a larger role. Our meta-regression findings support this: certain echocardiographic parameters were more predictive in specific patient subgroups. For instance, both TAPSE and RV/LV diameter ratio were most predictive in patients not on preoperative IV inotropes, while RVFAC was most predictive of RVF in patients not receiving preoperative IABP and those with higher (i.e., more stable) preoperative INTERMACS status. These findings suggest that predictors of RVF are more reliable in stable LVAD candidates, where measurements are less confounded by acute hemodynamic support such as temporary circulatory support, or deterioration. In such patients, echocardiographic values may more accurately reflect the intrinsic function of the RV and its reserve, thereby having stronger predictive ability. It is also important to acknowledge that although these parameters may be broadly predictive, the optimal thresholds for high-risk classification could vary between subgroups. For example, while lower RVFAC values might indicate heightened RVF risk across all patients, the specific cutoff for high-risk designation may differ depending on baseline characteristics such as INTERMACS status. Future research should potentially focus on refining subgroup-specific thresholds to improve predictive accuracy and reproducibility.

Furthermore, other sources of heterogeneity explored in this study include variation in LVAD technology over the 23-year study period –particularly the transition from earlier pulsatile-flow pumps to modern continuous-flow devices –and changing surgical techniques over time. Continuous-flow LVADs, unlike their pulsatile predecessors, are associated with increased leftward septal bowing, which can potentially distort RV geometry on echocardiography and strain-based imaging^55^. However, subgroup analyses restricted to studies including only continuous-flow LVADs showed no reduction in heterogeneity, and meta-regression analyses using year of publication as a surrogate marker for evolving surgical practice revealed no significant impact on effect sizes. These findings suggest the predictive performance of echocardiographic markers has remained relatively stable over time, despite changes in LVAD technology and perioperative care.

Overall, while variation in RVF definitions contributes modestly to heterogeneity, differences in clinical characteristics and patient selection appear to play a more substantial role. The persistence of residual heterogeneity likely reflects additional unmeasured factors, perhaps including geographic differences in protocols, variability in the timing of echocardiographic assessments, and interobserver variability in measurement technique. Although such factors are difficult to quantify using aggregate-level meta-analysis data, these findings underscore the importance of adopting standardized echocardiographic protocols for preoperative assessment in LVAD candidates to improve reproducibility and clinical applicability across centers.

### Limitations and future directions

Despite its strengths, this study has several limitations. Firstly, all included studies were observational, introducing potential for selection bias. While all studies achieved high Newcastle-Ottawa Scale scores (7–9), the observational design of the studies still carries risks of selection bias and unmeasured confounding variables. Secondly, several studies reported medians and ranges or interquartile ranges, where standardized conversion methods (as recommended by the Cochrane Handbook) were applied to approximate the mean and standard deviation^14^. However, these methods assume the data are approximately symmetrically distributed, potentially introducing estimation bias in studies with significantly skewed distributions^14^. Furthermore, echocardiographic assessments, particularly for right-sided parameters such as TAPSE, RVFAC, and RVEF, are highly dependent on operator experience due to the RV’s complex geometry, which therefore introduces interobserver variability. Inconsistent probe position, image acquisition quality, and institutional protocols may further reduce the reproducibility of these echocardiographic measurements across studies. This may have further contributed to the observed heterogeneity between studies, highlighting the need for standardized imaging protocols.

Moreover, subgroup analyses using only studies applying the most common RVF definition helped control for endpoint definition variability. However, there were still some minor variations. While some studies included prolonged vasodilators or inhaled nitric oxide as part of the RVF definition criteria, others did not, and therefore we have not completely ruled out definition variability. Another important limitation arises from the potential for competing risks, particularly early postoperative mortality, which may preclude the development of the RVF endpoint. As most primary studies did not account for such competing events, the true incidence of RVF may have been underestimated.

A key challenge for real-world application is the persistent heterogeneity observed in several of our meta-analyses. This variability reduces confidence in applying pooled thresholds universally across diverse clinical populations. Our meta-regression findings suggest that reliance on any single parameter or cutoff may lead to over- or underestimation of RVF risk, particularly in less stable patients. Consequently, risk stratification should integrate multiple echocardiographic measures with clinical context, rather than depend on a cutoff for an isolated marker. Until validated in large, prospective LVAD cohorts, these findings should be viewed as an adjunct to, rather than a replacement for, comprehensive clinical risk assessment.

Furthermore, all analyses including pooled effect size estimation, meta-regression and threshold determination were based on study-level aggregate data rather than individual patient-level data. Therefore, these analyses are susceptible to ecological fallacy. For example, associations between study characteristics (e.g., preoperative IV inotrope use or INTERMACS status distributions) and effect sizes observed at the cohort level may not translate to individuals. Future efforts must prioritize individual-patient data (IPD) meta-analyses to validate and optimize RVEDD and RVFWS thresholds within multivariable risk models. Such models –ideally built from prospectively collected, multicenter datasets –can then be benchmarked against established scores (e.g., EUROMACS-RHF, CRITT) and inform the development of composite risk tools, potentially enhancing predictive accuracy and clinical usability. Although we did not develop such a model in this present study due to the constraints of aggregate-level data, our findings offer a framework for model development in future IPD studies. Insights from these validation studies could be especially valuable in resource-limited settings where there may be restricted access to routine invasive hemodynamic investigations such as right heart catheterization, due to their invasiveness, cost, and technical demands^56^.

Finally, it is also important to consider emerging imaging technologies—such as 3D echocardiography—which may offer incremental predictive value over 2D and strain-based measures. Some studies have already demonstrated that 3D-derived metrics—including 3D-RVEF, RV shape indices (free wall and septal curvature), RV end-diastolic and end-systolic volumes, and 3D strain—are significant predictors of RVF^57–59^. These parameters may provide a more comprehensive assessment of RV geometry and function, given the RV’s complex shape and mechanics. Future research should assess whether 3D echocardiography and strain-based parameters consistently outperform conventional 2D measures when assessed in large, standardized LVAD cohorts. Nonetheless, 2D echocardiographic thresholds may remain the most practical for routine use, as they are already part of standard preoperative assessments in many centers and are likely cost-neutral. In contrast, strain and 3D techniques, while potentially more sensitive, require specialized software and expertise, which may limit their widespread adoption due to higher upfront costs^60^. Until broader validation and accessibility are achieved, conventional 2D parameters remain the most feasible tools for widespread RVF risk stratification.

## Conclusion

This meta-analysis identifies higher right ventricular end-diastolic diameter (RVEDD) and less negative right ventricular free wall strain (RVFWS) as the strongest and most reliable preoperative echocardiographic predictors of RVF following LVAD implantation, aligning with previous work. Right ventricular end-diastolic area (RVEDA) also emerged as a reliable and moderately strong predictor, though few studies have explored this parameter thus far. TAPSE shows more promise as a stronger predictor than previous findings, but limitations in reproducibility remain. The lack of a uniform definition of RVF only partially contributes to the current challenges with reproducibility, while changes in LVAD technology and surgical practices have had no significant impact. In contrast, patient-specific factors and clinical characteristics play a more significant role. We propose that cutoffs of RVEDD ≥ 41.5 mm and RVFWS ≥ −11.3% offer high sensitivity and specificity for predicting RVF, though we also highlight that these markers are less predictive of RVF in LVAD candidates who are less stable preoperatively (e.g., low INTERMACS status, requiring IV inotropes, requiring IABP). Ideally, future research should utilize multicenter data to tailor cutoffs for specific population and disease subgroups, which may guide us towards stronger predictors that are applicable across centers. Clinically, improved risk stratification is crucial for facilitating targeted optimization strategies and earlier surgical decisions, potentially reducing the burden of RVF in the LVAD recipients.

## Financial disclosure statement

This study received no funding from external sources. There are no conflicts of interest to declare.
